# Patients languishing in emergency departments: A descriptive analysis of mental health-related emergency department presentations in Australia between 2016-17 and 2020-21

**DOI:** 10.1177/10398562231195438

**Published:** 2023-08-15

**Authors:** Matthew Brazel, Stephen Allison, Tarun Bastiampillai, Stephen R Kisely, Jeffrey CL Looi

**Affiliations:** Academic Unit of Psychiatry and Addiction Medicine, School of Medicine and Psychology, 2219 The Australian National University, Canberra Hospital, Canberra, ACT, Australia;; Department of Psychiatry, The Canberra Hospital, Garran, ACT, Australia; and; Consortium of Australian-Academic Psychiatrists for Independent Policy and Research Analysis (CAPIPRA), Canberra, ACT, Australia; Consortium of Australian-Academic Psychiatrists for Independent Policy and Research Analysis (CAPIPRA), Canberra, ACT, Australia; and; College of Medicine and Public Health, 1065 Flinders University, Adelaide, SA, Australia; Consortium of Australian-Academic Psychiatrists for Independent Policy and Research Analysis (CAPIPRA), Canberra, ACT, Australia;; College of Medicine and Public Health, 1065 Flinders University, Adelaide, SA, Australia; and; Department of Psychiatry, 2541 Monash University, Clayton, VIC, Australia; Consortium of Australian-Academic Psychiatrists for Independent Policy and Research Analysis (CAPIPRA), Canberra, ACT, Australia;; School of Medicine, 1974 University of Queensland, Princess Alexandra Hospital, Woolloongabba, QLD, Australia; and; Departments of Psychiatry, Community Health and Epidemiology, Dalhousie University, Halifax, NS, Canada; Academic Unit of Psychiatry and Addiction Medicine, School of Medicine and Psychology, 2219 The Australian National University, Canberra Hospital, Canberra, ACT, Australia; and; Consortium of Australian-Academic Psychiatrists for Independent Policy and Research Analysis (CAPIPRA), Canberra, ACT, Australia

**Keywords:** emergency department, mental health, Australia, outcomes, services

## Abstract

**Objective:**

In the context of concerns regarding hospital access block, this paper provides a descriptive longitudinal analysis of mental health–related ED episodes in Australian public hospitals between 2016-17 and 2020-21.

**Method:**

We descriptively analysed Australian Institute of Health and Welfare data for mental health–related ED presentations, outcomes and 5-year trends for Australian public hospitals.

**Results:**

There were more than 300,000 Australian mental health–related ED presentations in 2020-21. Presentations increased by an average annual rate of 2.8% between 2016-17 and 2020-21, commonly involving first responder (police, paramedic) attendance. From 2016-17 to 2020-21, the average annual rate of mental health–related ED presentations receiving a triage category of resuscitation increased by 13.7%, emergency by 9.4% and urgent by 4.7%. 90% of MH-related ED presentations were completed within 14 h, which was longer than the 90^th^ percentile for all ED presentations (up to 8 h).

**Conclusions:**

Current mental health policies have not stemmed the rising tide of ED presentations. Mental health–related ED presentations are increasing in number and severity, likely due to health systemic and societal factors. Psychiatry patients stay longer in EDs than other patients. Healthcare reforms should be targeted to provide the best outcome based on principles of equity of access.

Australian mental health (MH) care has seen changes in service delivery and demand over time. Deinstitutionalisation, limited acute psychiatric beds, insufficient community-based MH services, destigmatisation and societal changes have resulted in a tenfold increase in the number of patients with mental illness attending emergency departments (EDs) between 1993-94 and 2002-03.^1^ For 2016-17, the Australasian College of Emergency Medicine (ACEM) found those presenting to EDs for an MH reason waited longer, had a longer overall ED length-of-stay (LOS) and were more likely to leave ED prior to their treatment being completed.^2^ Such increasing ED LOS is central to acute hospital access block, thereby disrupting the flow of patients through to dedicated inpatient care and the overall functioning of public sector hospital care.^3^ This study aims to expand on previous research^1,2,4^ by providing a descriptive analysis of the most recent national data on Australian public hospital MH ED activity and outcomes.

## Methods

We used the most recently available data (2020-21) including 5-year trends for MH-related ED data collected by the National Non-Admitted Patient Emergency Department Care Database (NNAPEDCD), published by the Australian Institute of Health and Welfare (AIHW).

## Results

There were 309,657 MH-related presentations to public EDs in 2020-21. These increased by an average annual rate of 2.8% between 2016-17 and 2020-21. The per capita rate of MH-related ED presentations increased by an average annual rate of 1.5% over the same period.

### Mode of arrival

In 2020-21, MH-related ED presentations were more likely to arrive by ambulance, air ambulance or helicopter (52.2% of total MH-related ED presentations) compared to all ED presentations (26.2%). They also arrived more often by using police/correctional services (6.1%) compared to all presentations (0.6%). Between 2016-17 and 2020-21, the average annual rate at which MH-related presentations to ED arrived by ambulance, air ambulance or helicopter increased by 3.9% and by using police/correctional services decreased by 5.6%.

### Triage

In 2020-21, the majority of MH-related ED presentations received a high triage categorisation, with 52.2% being categorised as urgent, 17.3% as emergency and 1.4% as resuscitation. Of the remainder, 25.9% were classified as being semi-urgent and 3.1% as non-urgent. Between 2016-17 and 2020-21, the average annual rate of MH-related ED presentations receiving a triage category of resuscitation increased by 13.7%, emergency by 9.4% and urgent by 4.7%. Semi-urgent presentations decreased by an average annual rate of 1.2% and non-urgent presentations decreased by 13.9% during the same time period. 99.4% of MH-related ED presentations in 2020-21 were emergency in nature, 0.4% were a planned return visit and 0.2% a pre-arranged admission.

### Demographics

The age groups with the highest rate of MH-related ED presentations per 10,000 population in 2020-21 were 18- to 24-year-olds (219.8), followed by those aged 85 and older (183.5) and 12- to 17-year-olds (180.0) (Table 1).

**Table 1.** 2020-21 rate of mental health–related and total emergency department presentations in Australian public hospitals, by patient age

Demographic group	Mental health ED presentations per 10,000 population	Total ED presentations per 10,000 population
0–11 years	35.8	9,484.9
12–17 years	180.0	3,129.4
18–24 years	219.8	3,764.5
25–34 years	164.8	3,267.0
35–44 years	157.2	2,895.1
45–54 years	131.3	2,885.3
55–64 years	81.2	2,866.4
65–74 years	61.5	3,340.5
75–84 years	95.2	5,077.7
85 years and over	183.5	7,691.3

### ED outcomes

In 2020-21, only 63.8% of patients presenting to Australian public hospital EDs for an MH-related reason were seen on time according to their triage category. Of these ED presentations, 35.9% resulted in admission, 56.7% departed without being admitted or referred to another hospital, 2.7% left of their own choice and 0.5% did not wait to be seen.

**Figure 1.**
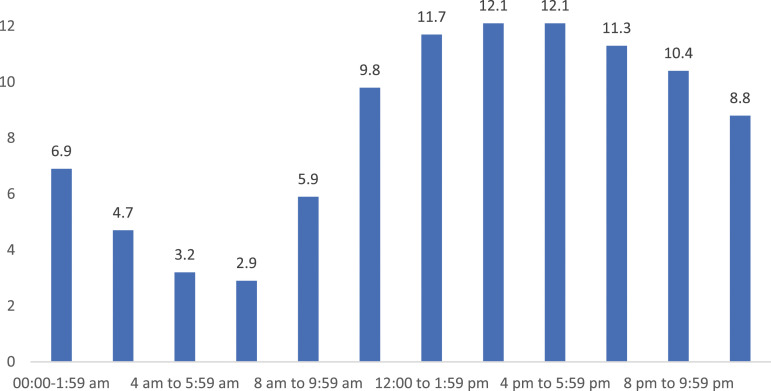
Proportion (%) of Australian 2020-21 public hospital mental health–related ED presentations by time of presentation.

**Figure 2.**
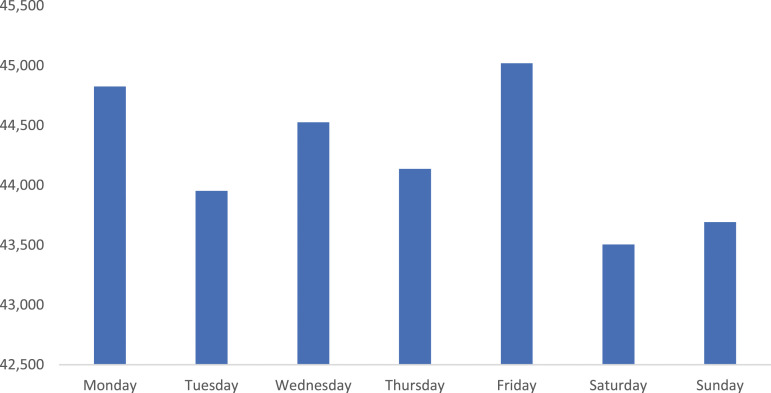
2020-21 mental health–related ED presentations to Australian public hospital EDs by day of presentation.

More MH-related ED presentations occurred in the afternoon and early evening (Figure 1) compared to other times of day. The number of presentations in 2020-21 was largely consistent across the days of the week, with Friday recording the greatest number of presentations (45,019), with Saturday having the fewest (43,505) (Figure 2).

The median length-of-stay for all MH-related ED presentations was 3h:57 m, and 5h:26 m for presentations ending in admission. 90% of MH-related ED presentations were completed within 13h:57 m, which was much longer than for all ED presentations (up to 8 h) (AIHW 2022a).

### Psychiatric diagnoses (ICD-10 codes)

The most common principal diagnosis for MH-related ED presentations in 2020-21 were neurotic, stress-related and somatoform disorders (26.9%), followed by mental and behavioural disorders due to psychoactive substance use (26.7%) (Table 2).

**Table 2.** Total number, proportion (%) of 2020-21 Australian public hospital mental health–related ED presentations by principal diagnosis and average annual change (%) between 2016-17 and 2020-21

Principal diagnosis	Number of 2020-21 ED presentations	Proportion (%) of total 2020-21 mental health–related ED presentations	Average annual change (%) 2016-17 to 2020-21
Organic, including symptomatic, mental disorders	26,081	8.4	6.0
Mental and behavioural disorders due to psychoactive substance use	82,560	26.7	2.6
Schizophrenia, schizotypal and delusional disorders	35,808	11.6	3.1
Mood (affective) disorders	28,923	9.3	−2.5
Neurotic, stress-related and somatoform disorders	83,306	26.9	3.1
Behavioural syndromes associated with physiological disturbances and physical factors	6,016	1.9	23.9
Disorders of adult personality and behaviour	6,067	2	−5.3
Disorders of psychological development	600	0.2	21.2
Behavioural and emotional disorders with onset usually occurring in childhood and adolescence	9,168	3	5.9
Mental disorder, not otherwise specified	31,110	10	4.0

Between 2016-17 and 2020-21, there were average annual increases of 3.1% for both schizophrenia, schizotypal and delusional disorders, and stress-related and somatoform disorders as principal diagnoses for MH-related ED presentations. Substance-related disorders as principal diagnoses increased by an average annual rate of 2.6%, but there was a reduction in the number of affective disorders (−2.5%) and disorders of personality and behaviour (−5.3%) over the same period (Table 2).

## Discussion

Continuing a trend identified in earlier research,^2^ Australian MH-related ED presentations increased between 2016-17 and 2020-21. The cause for this is likely multifactorial. Mental illness is increasingly prevalent in Australia, leading to increased MH-related ED presentations.^5^ This, combined with barriers to primary care, insufficient community MH care services^6^ and limited inpatient psychiatric beds,^7^ has possibly left ED as the only avenue for many to seek care. Awareness campaigns by advocacy groups such as Beyond Blue and headspace, as well as crisis and after-hours referrals from such services, may have led to increased service demand. There is also a possibility that increased social media use may have contributed to rising ED mental health–related presentations particularly for young women.^8^

Australians presenting to ED for MH-related problems increasingly arrived by ambulance services. This signifies a potentially significant unmet need for urgent community MH care, likely due to chronic underfunding of state and territory-based MH services.^9^ Increased training and initiatives such as Police/Ambulance Clinical Emergency Response (PACER) teams may partially explain the reduction in the police/correctional service involvement in presentations.

Triage categorisation for MH-related ED presentations was high during 2020-21, demonstrating that patients seeking emergency care are in substantive need of urgent assessment. By the time the patients presented, complicating factors such as deliberate self-harm may have already occurred, increasing the triage category.

36.2% of MH-related ED presentations were not seen in the recommended timeframe required by their triage category, and patients with MH-related ED presentations were more likely to have extended length-of-stay, increasing the risks of adverse experiences due to long stays in overstimulating ED environments. These longer stays are likely due to insufficient MH expertise available within EDs, either locally or provided via consultation, and likely due to a shortage of inpatient beds for patients requiring admission.^6,10^ Improved ED resourcing during times of peak demand such as afternoons and evenings may enhance access for these patients.

The highest rates of MH-related ED presentations were for adolescents, young adults and the oft-forgotten elderly. This possibly reflects high prevalence of MH problems within these populations, combined with limitations in child and adolescent,^11^ youth^12^ and older persons specific MH services.^13^ Dementia is a common condition in those 85 years and older,^14^ and its neuropsychiatric symptoms may contribute to the high rates of MH-related ED presentations for the elderly.

Psychotic disorders, neurotic, stress-related and somatic disorders all increased in frequency as principal diagnoses of ED presentations while the rates of affective disorders decreased. Efforts to upskill general practitioners (GPs) in affective disorders may have increased primary care access and caused a corresponding decrease in ED presentations. Perhaps primary care doesn't address well the needs of those with psychotic, anxiety/stress and substance-related disorders. The development of training programs for GPs, as well as enhanced and sustainable numbers of GPs, together with psychological therapy access, may potentially reduce MH-related ED presentations.

MH-related ED presentations for disorders secondary to substance use increased during the recorded time period, matching findings from previous research.^4^ This could possibly be explained by a continued growth in methamphetamine use,^15^ a substance linked to increasing MH-related ED presentations,^4,16^ and long-term psychotic disorders.^17^ The COVID-19 pandemic may have also increased the need for urgent care in those with substance use disorders,^18^ increasing ED presentations. This complex health and social issue would benefit from a national strategy for substance use disorders, as well as implementation of guidelines and appropriately resourced services that link to primary care.

## Limitations

AIHW data may not accurately capture variations in service structure and definitions across different Australian health jurisdictions. Psychiatric diagnoses are longitudinal, and as such, cross-sectional principal ED diagnoses may not accurately reflect the true nature of a patient’s diagnosis at the end of their episode of care, which may often include significant comorbidities such as underlying personality disorders. These may not be captured in administrative health data that are restricted to broad primary diagnostic categories and may also be subject to recording bias. Triage categorisation may vary across sites in accordance with local training and policy. Treatment may have been provided in other settings, such as in urgent psychiatric care clinics, or step up/step down programs, and so would not be accurately reflected in AIHW data. AIHW data does not definitively allow for the reason behind the changes in MH-related ED presentations to be established.

## Future directions

Better resourcing, infrastructure and staffing of both acute hospital and community MH care^19^ could reduce the need for emergency MH presentations. This should be matched by better access to private sector outpatient^20^ and inpatient^21^ Medicare-subsidised MH services, especially primary care underpinned by GPs. Epidemiologically based health needs assessments and mapping of services are needed to ascertain the mental healthcare terrain. Development of more sophisticated outcome measures will help inform evaluation of service efficacy. A coordinated national approach to the management of substance use disorders may improve outcomes and reduce MH-related ED presentations. We also need to understand why there have been significant increases in ED mental health–related presentations despite increased investment in Medicare-subsidised MH services,^20^ increased use of psychotropics (antidepressants) and whether this may also relate to underlying secular changes within society. Finally, we acknowledge that the other elements of the quality and LOS public sector inpatient care^22^ and hospital exit block^23^ are also factors that warrant separate future research. Future studies could correlate national AIHW data with individual state/territory/statistical area level 3 measures, where available, to permit finer-grained understanding of local variations that may impact on the data. EDs are an essential component of a safe and effective MH system, and those with mental illnesses have the needs and rights to the same level of care as those presenting for other reasons.
